# Application of the new at-column dilution (ACD) modulator for the two-dimensional RP×HILIC analysis of *Buddleja davidii*

**DOI:** 10.1007/s00216-020-02392-3

**Published:** 2020-01-21

**Authors:** Yingzhuang Chen, Lidia Montero, Jiang Luo, Junjie Li, Oliver J. Schmitz

**Affiliations:** 1 Applied Analytical Chemistry, University of Duisburg-Essen, Universitaetsstr. 5, 45141 Essen, Germany; 2 Key Laboratory of Phytochemical R&D of Hunan Province, Key Laboratory of Chemical Biology & Traditional Chinese Medicine Research, Ministry of Education, Hunan Normal University, Changsha, 410081 Hunan China; 3 Teaching and Research Center for Separation (TRC), University of Duisburg-Essen, Universitaetsstr. 5, 45141 Essen, Germany

**Keywords:** RPLCxHILIC, At-column dilution (ACD), Herbal medicine, 2D-LC, *Buddleja davidii*

## Abstract

The focus of this study was the analysis of the complex chemical composition from different parts of *Buddleja davidii*, whose species are commonly known as ornamental plants and herbal medicines in many countries. As an herbal medicine, it has been utilized for stroke treatments, headache, wound healing, neurological disorder, etc. However, the understanding of its chemical matrices is still insufficient. Therefore, an online two-dimensional reversed phase liquid chromatography x hydrophilic interaction liquid chromatography (RPLCxHILIC) system coupled with mass spectrometry was applied for further detailed investigation of the chemical constituents in *Buddleja dividii*. In this two-dimensional liquid chromatography (2D-LC) method, a new at-column dilution (ACD) modulator was introduced in the 2D-LC system to solve the incompatibility problem of the mobile phase between two dimensions, which resulted in a 2D-LC analysis with high orthogonality. For the root extract, as one of the analyzed samples, the optimization of the ^1^D and ^2^D gradients was carried out carefully. With this new modulator, much better peak separation and better peak shape were achieved compared to two-dimensional liquid chromatography system using a traditional standard (TS) modulator. With a similar approach, the other four parts of *Buddleja davidii* were well separated. Comparing the different analyzed parts, flowers and leaves showed the most complex profiles. MS and MS/MS data were obtained successfully, which demonstrated the potential of the proposed RPLCxHILIC-MS system in the constituents’ analysis of herbal medicine. However, due to the lack of reported reference information, 24 compounds could be tentatively identified.

**Electronic supplementary material:**

The online version of this article (10.1007/s00216-020-02392-3) contains supplementary material, which is available to authorized users.

## Introduction

In recent decades, as a rising star in the field of chromatography, two-dimensional liquid chromatography (2D-LC) has attracted more and more attention by rapid developments in research of complex systems, such as natural products, environmental pollutants, food and pharmacy, proteomics, and metabolomics [1]. The largest benefit of 2D-LC is the dramatic increase in peak capacity that may be achieved, which is reflected in the component overlapping reduction [2].

In online comprehensive 2D-LC (LCxLC), where the sample is completely and continuously transferred from the first dimension (^1^D) to the second dimension (^2^D), the combination of orthogonal dimensions is usually hard to achieve because incompatible mobile phases are often used in both dimensions. For example, one of the most orthogonal combinations that can be used in 2D-LC is the combination of RPLC and HILIC because these two separation mechanisms present completely different selectivity for the retention of the analytes. Besides, the mobile phases used in RPLC and HILIC are miscible and compatible with detectors such as MS. Therefore, a 2D-LC system based on the coupling of RPLC and HILIC (HILICxRPLC or RPLCxHILIC) should theoretically be a promising system [1]. However, the compatibility of the mobile phases in such two dimensions is always a problematic and an important issue. The main reason is that the weak solvent in the ^1^D consists of a strong eluent solvent for the ^2^D, which can lead to a problem of mobile phase mismatch and poor or no separation in the ^2^D.

In particular, the analysis of polar compounds by an RPxHILIC coupling has been substantially less employed than the HILICxRP combination in the literature so far. The difficulty arises from the high HILIC sensitivity to the injection solvent, fractions coming from the ^1^D-RP solved in a high aqueous concentration are hardly focused in the ^2^D-HILIC causing a breakthrough phenomenon due to the non-retention of the transfer compounds in the ^2^D column. This effect is even more negative when the injection volume exceeds the 9% of the column dead volume [3]. However, HILIC is able to provide fast analysis of compounds with a low loss of resolution [4] that can be a very interesting characteristic for the separation carried out in the ^2^D. So, the fast analysis provided by HILIC could be extrapolated to the analysis of polar compounds, which 2DLC analysis was mainly limited to the coupling of HILIC in the ^1^D and RP in the ^2^D up to now.

A big effort should be done in this kind of orthogonal coupling to avoid the mismatch solvent effect as far as possible. As a core part of a 2D-LC system, the modulator is responsible for the ^1^D effluent cutting, collecting, and transferring to the ^2^D column. The incompatibility of two dimensions will negatively affect the modulation process, resulting in analytes unfocussed on the head of the ^2^D column. The tradition standard modulation (TS) is the most common and simple way to transfer the fractions from the ^1^D to the ^2^D, and consists on the use of two identical injection loops installed in a two-position switching valve. These two loops continuously alternate their functions collecting the fractions from the ^1^D and injecting them in the ^2^D column. This modulation mechanism can be considered as a passive modulation since the fraction injected in the ^2^D column is going to depend only on the ^1^D flow rate and mobile phases. Due to this passive modulation, usually, the TS modulation produces a low focusing effect in the head of the ^2^D column, because it is not able to reduce the strength of the fraction solvent before reaching the ^2^D column.

Recently, several kinds of modulators have been developed to overcome the incompatibility problem [5]. Some of them are based on the modification of the modulator configuration like the Fixed Solvent Modulator (FSM) developed by Petersson et al. [6] or the Active Solvent Modulation (ASM) proposed by Stoll et al. [7, 8].

Besides the solvent incompatibility, LCxLC presents other limitations. For instance, due to the realistic limitation of the transferred volume from the ^1^D to the ^2^D, ^1^D column diameter and flow rate are usually low in LCxLC and a small fraction is collected in the modulator which will be diluted by the ^2^D high flow rate. Therefore, these limitations result in a hard detection of low abundant targets, i.e., low sensitivity. Based on the FSM modulation strategy, Chen’s group made a new development to improve the ^2^D detection sensitivity by replacing the sample loops with short C18 trapping columns to construct an RPLC×HILIC system [9], which is shown in Fig. S1 in the Electronic Supplementary Material (ESM). However, in all three described modulators, the fraction dilution factor depends on the length and inner diameter of the sample loop channel and the bypass channel or the length of the trapping and the splitting column. This fact involves precise control and optimization of the dilution factor during the analysis of a complex sample containing analytes with a wide range of polarity which can limit the method development.

**Fig. 1:**
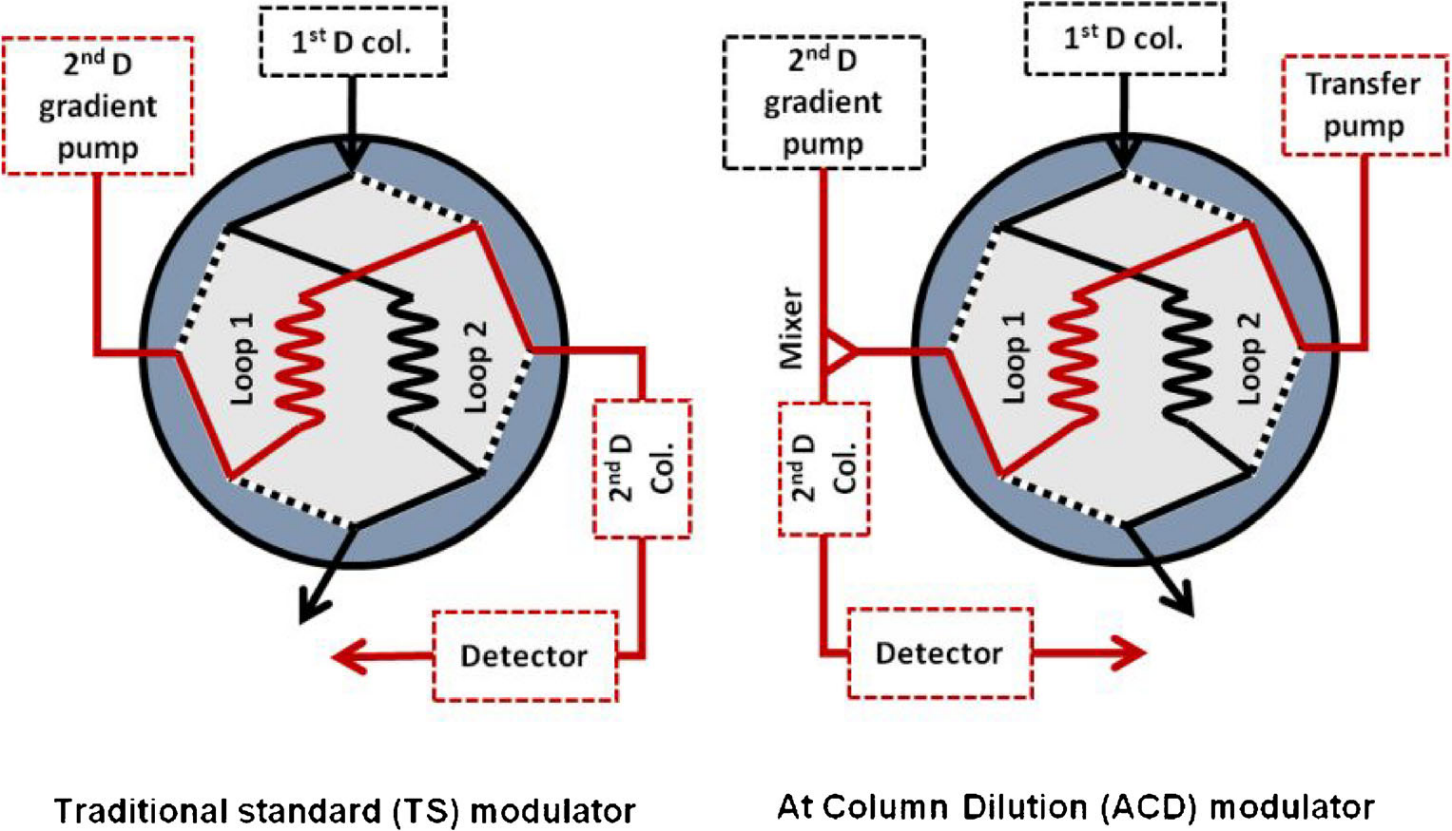
Configuration of the traditional standard (TS) modulator and at-column dilution (ACD) modulator. In this scheme, a 2-position 8-port switching valve is shown to simplify the visualization of the new configuration

As a further improvement, a novel modulator, called at-column dilution (ACD) modulator, was proposed in our previous work to achieve free adjustment of the online dilution factor [10]. The RPLCxHILIC configuration equipped with an ACD modulator is shown in Fig. 1. The ACD modulator was modified from the TS modulation (Fig. 1) by adding an independent transfer pump, which is responsible for the injection of the fractions into the ^2^D. In this modulation strategy, the ^2^D mobile phase is no longer flowing through the sample loops, but the additional transfer pump is connected to the valve, and therefore, it is the responsible for eluting the ^1^D fraction storage in the loop with a weaker solvent for the ^2^D (transfer flow rate). Afterward, the transfer flow and the ^2^D gradient flow meet and are mixed in a mixer before reaching the ^2^D column. Thereby, the high water content fraction from the ^1^D-RP is diluted with acetonitrile and gradually enters the ^2^D column, and the analytes can be focussed on the head of the ^2^D column. More importantly, the dilution factor could be controlled by adjusting the transfer flow and the ^2^D flow rates, since the transfer flow can be optimized regardless of the ^1^D and ^2^D conditions.

Herbal medicines are naturally occurring plant-derived substances, which are used for medical purposes [11]. According to the World Health Organization (WHO), about 80% of the world’s population uses herbal medicines as its primary healthcare [12]. There is no doubt that herbal medicines have great medical potential and economical importance. However, the difficulty of obtaining a clear description of the complex chemical constituents limits the in-depth study of the efficacy principles and the quality control of these herbal medicines in practice. Therefore, the development of analytical methods and tools that allow the comprehensive analysis of the herbal medicines is highly demanded. However, due to the high complexity of the chemical constituents in herbal medicines, conventional one-dimensional chromatographic techniques usually do not provide enough separation power to comprehensively analyze the chemical profile of such complex plants. Therefore, there is a motivation to seek more advanced analytical tools with higher separation performance.

*Buddleja davidii* is a multi-stemmed shrub or small tree that is native to China and has been introduced nowadays as an ornamental plant worldwide, both in sub-oceanic climates and sub-Mediterranean zones. In Germany, *Buddleja davidii*, commonly known as the butterfly bush, has very wide distribution due to its small wind-dispersed seeds rapidly colonize and barely disturb the area. It also grows widely due to its potential invasive power and adaptability.

Similar to other species of *Buddleja*, *Buddleja davidii* has significant pharmaceutical activities related to its metabolites. For example, terpenes (ESM Fig. S2a), phenylethanoids as verbascoside, shown in ESM Fig. S2b, which is known for its antioxidant, anti-inflammatory and photoprotective actions [13], and phenylpropanoid glycosides (ESM Fig. S2c), with antioxidant activities, also isolated and identified in this plant [14–16]. A flavone glycoside called linarin, shown in ESM Fig. S2d, was also isolated, and this compound revealed analgesic, antipyretic, anti-inflammatory, and neuroprotective activities [17]. Furthermore, a systematic survey of the genus *Buddleja* has indicated that some isolated compounds have anti-inflammatory and inhibitory activity against eicosanoid synthesis by inhibiting cyclo-oxygenase (COX) or 5-lipoxygenase (5-LOX) [15, 18]. Therefore, a comprehensive study about its chemical constituents is highly required due to its rich and diverse potential medicinal value.

In this study, the applicability of ACD modulator is tested for the coupling of RPLCxHILIC-MS system. For this propose different parts of the herbal medicine *Buddleja davidii* were used as an example for the method development. As a starting point, careful optimization of ^1^D and ^2^D gradient separation was carried out for the root extract. Then, with the optimized 2D-LC separation conditions, four parts of *Buddleja davidii* were also analyzed. Finally, the 2D-LC-UV method was coupled to MS, and MS/MS experiments were carried out to identify the complex profile of the root sample.

## Experimental

### Chemicals

Acetonitrile (ACN, MS grade) was purchased from VWR International (USA). Formic acid (HPLC/SPECTRO grade) was from Merck KGaA (Germany) and ethanol (Analytical reagent grade) was purchased from Fisher Chemical (Fair Lawn, New Jersey, USA). Distilled water was filtered through a Sartorius Stedim Biotech system (Germany).

### Sample collection and preparation

The whole *Buddleja davidii* plant was collected from the field around the University of Duisburg-Essen, Campus Essen, Germany. The whole plant was divided into five parts: roots, stems, leaves, flowers, and fruits. The roots were washed with distilled water and dried with a dryer at 45 °C during 12 h. The other four parts were directly dried in a dryer at 45 °C. Then, they were smashed into powder and preserved in brown glass bottles at room temperature. 5.0 g of each sample were extracted with 50 mL 60% ethanol for 1 h at room temperature in glass flasks, and then the samples were extracted in an ultrasonic bath (VWR, USA) for another 1 h. After that, the samples were centrifuged (5840R Eppendorf, Germany) at 4000 rpm/min for 15 min at room temperature. The supernatant of the extract was moved to an ultracentrifuge (Spin plus from Eppendorf, Germany) and centrifuged at 14000 rpm/min for 10 min at room temperature. Afterward, 1 mL volume of the supernatants was diluted with 4 mL distilled water and the sample solution was filtered through a 0.2-μm filter prior to 2D-LC analysis.

### Two-dimensional liquid chromatography (2D-LC) system

An Agilent 1260 Infinity (^1^D) combined with Agilent 1290 Infinity (^2^D) was utilized as a 2D liquid chromatography system (Agilent Technologies, USA) in this study. In particular, the ^1^D was built by a 1260 Infinity Flexible Cube solvent management module, a 1260 Infinity Degasser, a 1260 Infinity Capillary Pump, a 1260 Infinity Micro Autosampler, a 1260 Infinity Thermostat Column Compartment, a 1260 Infinity Diode Array Detector. The ^2^D consisted of a 1290 Infinity II Binary Pump and a 1290 Infinity II Diode Array Detector. The interface or modulator consisted of a 1290 Infinity Valve Drive and a 2-position/4-port duo-valve equipped with two 80 μL loops. The third pump, used for the ACD modulation was a 1290 Infinity binary pump. The 2DLC system was controlled by the OpenLab CDS software. The data of the diode array detector were collected during the running of the system and then imported to LC Image 2.7r LC × LC (64-bit) to generate 2D contour plots. The detection wavelength was set at 280 nm during the whole separation process.

The configuration of the RPLCxHILIC-MS system with the ACD modulator is shown on the right side of Fig. 1. At first, the mobile phase from the ^1^D pump transferred the injected sample (2 μL) to the RP-^1^D column. The ^1^D effluent was alternatively stored in the sampling-loops of the electronically controlled 2-positon/4-port duo-valve, which hyphenated the ^1^D column and the ^2^D column. The switching time of the valve was set at 2 min (modulation time). Once the sample loop was filled with ^1^D effluent, the valve changed the position and the fraction was connected in the line of ^2^D flow, and then the collected fraction was pushed out of the sample loop by the transfer flow from the additional pump (transfer pump). At downstream of the modulator, the transfer flow—with a relative low flow rate—and the ^2^D eluent—with a high flow rate—met and mixed in a mixer and were transferred into the ^2^D column. Finally, after the detection in the ^2^D UV-detector, the ^2^D effluent was split to enter into the MS. In this work, the optimized transfer flow rate was 0.2 mL/min and the ^2^D flow rate was established at 2.4 of mL/min, which lead to a total ^2^D flow rate of 2.6 mL/min and a dilution factor of 13 (total flow rate/transfer flow rate).

**Fig. 2:**
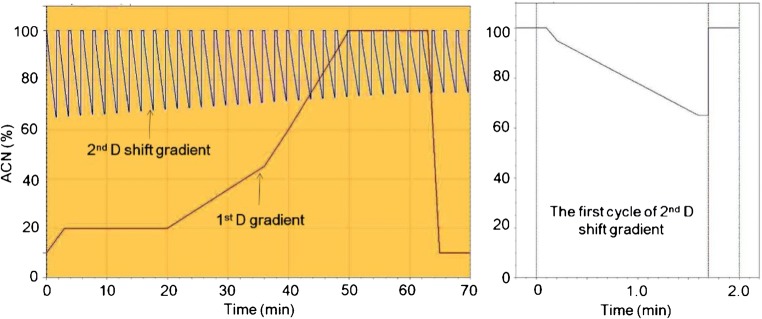
Optimized 2D-LC gradient for ^1^D and ^2^D separation

^1^D RP separation was performed at room temperature on a C18 column (150 × 1.0 mm) with 2.6 μm core-shell particles (Phenomenex, CA, USA). The mobile phase contained water (A) and acetonitrile with 0.1% formic acid (B). The optimized gradient time was 70 min at a flow rate of 15 μL/min. The gradient consisted of: 0 min, 10%B; 3 min, 20%B; 20 min, 20%B; 36 min, 45%, 50 min, 100% B; 64 min, 100% B; 65 min, 10% B (Fig. 2).

^2^D HILIC separation was performed at room temperature on a Cys-HILIC column (150 × 3.0 mm) packed with 5 μm particles (Acchrom, Inc., Beijing, China). The mobile phase contained water with 0.1% formic acid (A) and acetonitrile (B). The modulation time was 2 min, which leads to 35 cycles presented in the 2D separation. As shown in Fig. 2, the lowest point of the ^2^D shifting gradient in each cycle increases in 70 min from 65 to 75% of acetonitrile (B). The gradient was established as follow: 0 min, 100% B; 0.1 min, 0.1% B; 0.2 min, 95% B; 1.5 min, 75% B (at the beginning) or 65% B (at the end); 1.6 min, 75% B (at the beginning) or 65% B (at the end), 1.7 min, 100% B; 100% was maintained until 2 min for the equilibration of the column before the start of the next measurement.

### Mass spectrometry

Mass spectrometry was performed on an Agilent 6545 Q-TOF LC/MS (Agilent Technologies, USA) using an electrospray ionization source (ESI) operating in negative ion mode. A flow of 0.4 mL/min was introduced into the ion source of the MS by splitting the ^2^D flow rate with a T-valve. Nitrogen was utilized as sheath gas and drying gas. The sheath gas and the drying gas were set to 11 L/min at 300 °C and 10 L/min at 350 °C, respectively. The nebulizer was set at 35 psig and the capillary voltage and nozzle voltage were set at 3500 V and 1000 V, respectively. The fragmentor, skimmer, and Oct 1 RF Vpp were set at 220 V, 65 V, and 750 V, respectively. The acquisition mode was performed on full MS and auto MS/MS. The acquisition was performed in full scan and automatic MS/MS, at 1 spectra/s, in the *m*/*z* range 100–1500 (MS) or 100–1300 (MS/MS). The collision energy was 40 eV. The MS system was controlled by the software Agilent Mass Hunter Workstation Data Acquisition. Then the data could be analyzed by Agilent Mass Hunter Qualitative Analysis Navigator and imported into LC Image 2.7r LC × LC (64-bit) to generate MS contour plots.

## Results and discussion

### Optimization of the gradient for the ^1^D (RPLC) separation

**Fig. 3:**
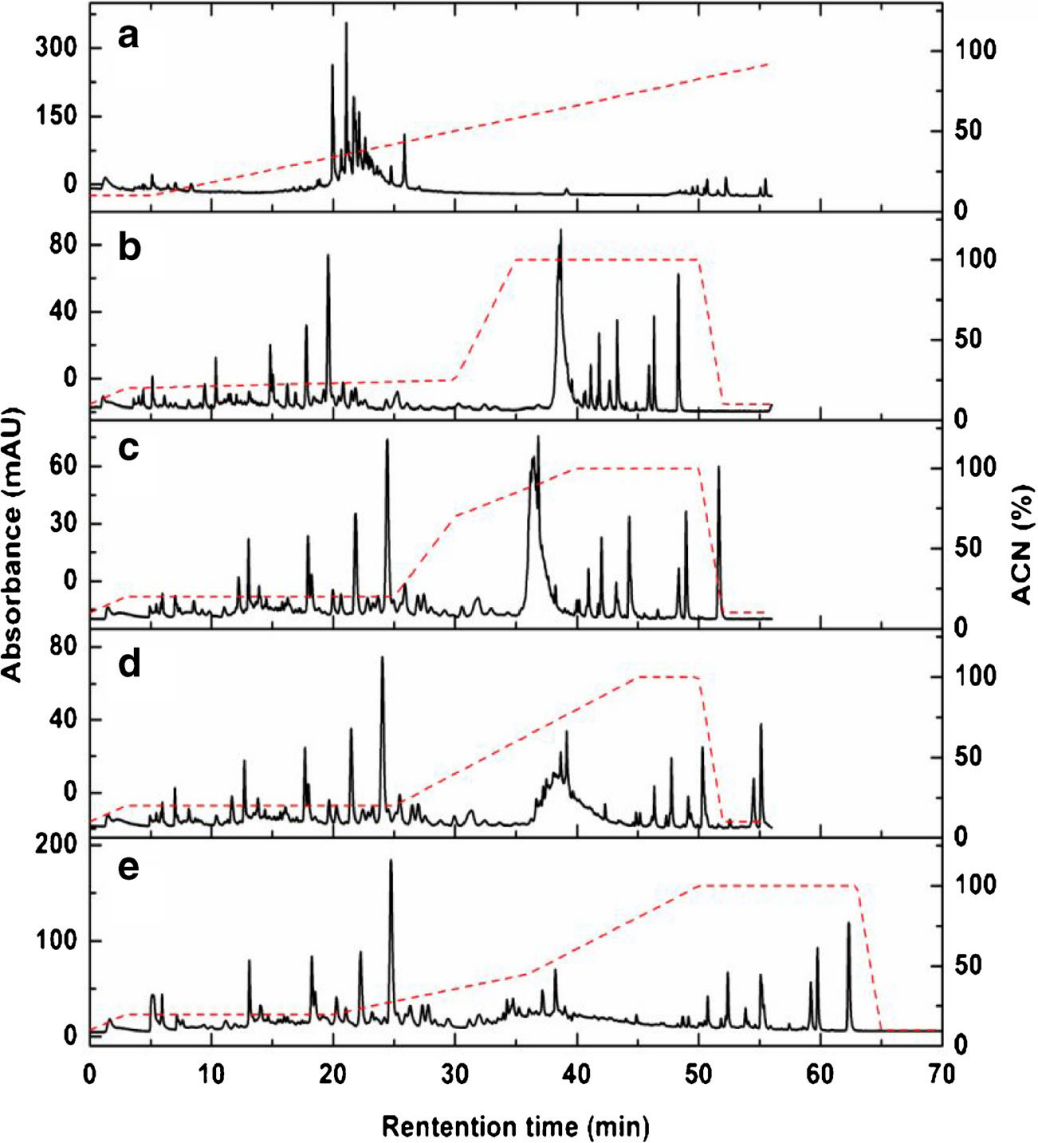
Optimization of the gradient for the ^1^D RP separation

The different parts of *Buddleja dividii* were expected to present similar metabolite families, therefore, the root was chosen to optimize the ^1^D RP separation gradient. Several gradients were tested, the detailed information about these gradients is listed in ESM Table S1. Figure 3 shows the chromatograms of the root extract separation obtained with the listed gradients. At first, to measure the polarity distribution of all constituents, a full gradient with ACN as organic solvent from 5 to 90% in 55 min was performed as shown in Fig. 3a. Most of the peaks were overlapped without being well separated in the time interval between 20 and 25 min. To perform a better separation of all compounds, a de novo optimization strategy was applied. In this strategy, the optimization was consequently performed segment by segment from the beginning. When the separation of analytes in a segment was optimized, the analytes in the next segment were eluted directly with a high content of organic phase to save time. By analogy, the optimized segment was fixed and the next segment continued to be optimized until the separation optimization of all analytes was completed (Fig. 3a–e). As shown in Fig. 3e, after only four steps, an evenly distributed separation was achieved with a final analysis time of 70 min. Therefore, the gradient showed in Fig. 3e was applied in the following experiments as an optimized ^1^D gradient.

### Optimization of the gradient for the ^2^D (HILIC) separation

The composition of mobile phase in the early stage of ^2^D gradient cycle is critical for focusing the transfer analytes on the head of the ^2^D column. Any modification of the ^2^D gradient in this stage might change the separation in ^2^D. Therefore, the optimization of the ^2^D HILIC gradient will focus on the early stage of the gradient cycle.

In this term, as a first and common strategy, we installed 80 μL loops in the interface. Considering the ^1^D flow rate (15 μL/min) and the modulation time (2 min), loops of 30 μL volume would be enough for the collection of the whole ^1^D fraction; however, with the use of larger loops (80 μL) connected to the interface, 50 μL of the loops was filled with ^2^D mobile phase, which allowed diluting the ^1^D fraction in a more adequate solvent, as demonstrated by Montero et al. [19]. This fact contributed to reduce the mismatch solvent incompatibility together with the ACD modulation, helping the focusing effect in the head of the ^2^D column.

In this regard, the effect of the injection volume in relation to the column void volume of the ^2^D column is a critical point in online LCxLC, mainly due to the combination of the ^1^D solvent strength and the very fast flow rates typically used in the ^2^D may hamper the performance of the separation of the analytes solved in the injected fraction, producing a band-broadening effect. Typically, the injection volume in the ^2^D column (loop size) should not exceed the 15% of the column void volume to reduce the band-broadening negative effects [20]; however, in the case of HILIC, the recommendation is much stricter considering the important effect of the injection solvent on the HILIC separation. Some results recommend that the injection volume should not exceed the 9% of the column void volume [3] but others reduce this percentage until the 1% [21]. In this work, when the TS modulator was employed, the injection volume (80 μL) implied the 11% of the ^2^D column volume, considering the dimensions of the ^2^D column (150 × 3 mm, 5 μm) and the interstitial column volume (68% of the total volume), which determine a dead volume of the ^2^D column of 720.8 μL. However, with ACD modulation, there is not a real injection volume as it happens in the TS modulation, since in ACD the transfer pump elutes the fraction from the loop with a controlled flow rate (slower than the ^2^D pump flow rate used in the TS modulator configuration for the elution of the fraction collected in the loop) and after that, this flow rate is mixed with the high ^2^D flow rate, so in this case, we can talk about a transfer fraction diluted by two different flow rates more than an injection volume, which improves even more the focusing effect in the head of the ^2^D column.

**Fig. 4:**
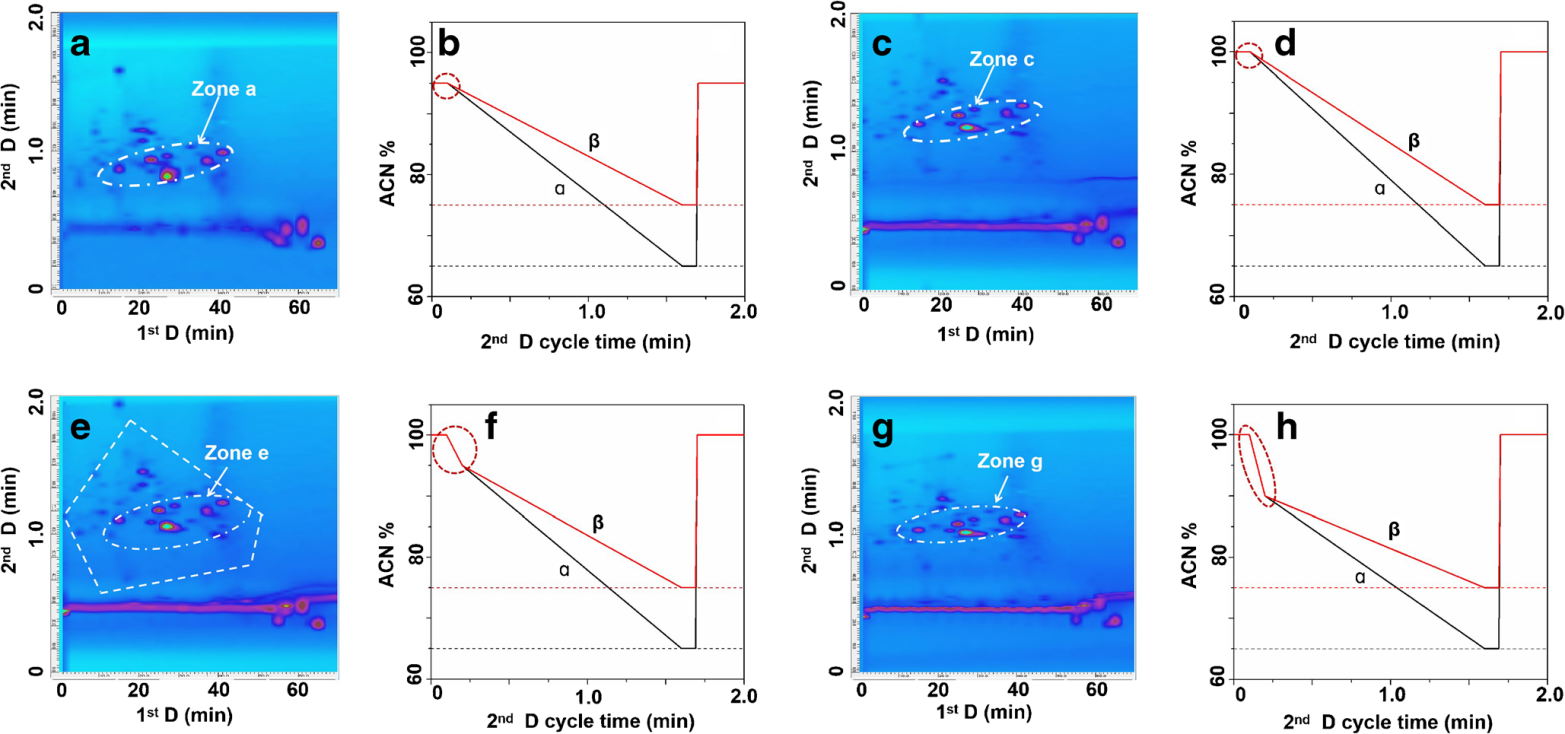
Optimization of the ^2^D shift gradient (**α**, first gradient cycle and **β**, last gradient cycle in ^2^D shift gradient). **a** 2D plot of the root separation using the gradient shown in **b** (95% of mobile phase B as initial conditions in the ^2^D gradients). **c** 2D plot of the root separation using the gradient shown in **d** (100% of mobile phase B as initial conditions in the ^2^D gradients). **e** 2D plot of the root separation using the gradient shown in **f** (100% of mobile phase B followed by a second step reducing the mobile phase B as initial conditions in the ^2^D gradients). **g** 2D plot of the root separation using the gradient shown in **h** (100% of mobile phase B followed by a second step with a faster reduction of mobile phase B as initial conditions in the ^2^D gradients)

Moreover, the use of shifting gradients along the LCxLC analysis not only helps to achieve more orthogonality but also to reduce the breakthrough since a modification of the ^2^D initial mobile phase conditions can be set accordingly to the ^1^D eluent. Fig. 4b, d, f, and h show four different ^2^D shift gradients, in which α and β represent the first gradient cycle and the last gradient cycle, respectively in the ^2^D shift gradients. In Fig. 4a, c, e, and g, the corresponding separations are shown. At the beginning and the end of the 4b and 4d gradients, the organic content of ACN was set at 95% and 100%, respectively. The separations obtained using these two gradients are shown in Fig. 4a and c. Although theoretically, under HILIC mode separation a small aqueous percentage is needed to create a water-layer around the polar particles, comparing both conditions, using 100% ACN as initial conditions the retention of the compounds was significantly increased (Fig. 4d). In addition, the peak width in zone c (Fig. 4c) was much narrower than that in zone a (Fig. 4a). This indicates that a 100% ACN used at the beginning and at the end of the fast ^2^D gradient leads to a better separation. Applying higher percentage of ACN at the end of the gradient would accelerate the equilibrium of the ^2^D HILIC column and prepare the conditions for the injection of the next cycle. Furthermore, applying higher content of ACN at the beginning of the gradient would improve the dilution effect of transferred fractions under ACD modulation mode, which results in better focus of analytes on the head of the ^2^D HILIC column. However, as can be observed in Fig. 4c, the separation with the gradient 4d was not optimal since the main part of the peaks were separated within a narrow small ^2^D area. The adjustment of the gradient was performed based on the gradient shown in Fig. 4d as shown in Fig. 4f and h. A rapid decrease of ACN to 95% (Fig. 4f) or 90% (Fig. 4h) was set in each cycle between 0.2 and 0.25 min. The chromatogram shown in Fig. 4f indicates that 5% decrease of ACN resulted in a slightly higher coverage of the 2D space in comparison with the distribution achieved using gradient 4h.

It is worth to point out that serious breakthrough was observed in the 2D contour plot. This might be attributed to a large amount of low polar compounds in the roots, which were hardly retained on the ^2^D HILIC column. Nevertheless, any combination would have limitations. In this case, the combination of RPLC/HILIC is appropriated for the separation of amphiphilic compounds but is not suitable enough for the separation of homophilic compounds. The target of this work would focus on the amphiphilic compounds rather than homophilic compounds. Therefore, gradient 4f was determined as the optimal ^2^D gradient used for the LCxLC system for the following measurements.

### RPLCxHILIC for different parts of *Buddleja davidii*

**Fig. 5:**
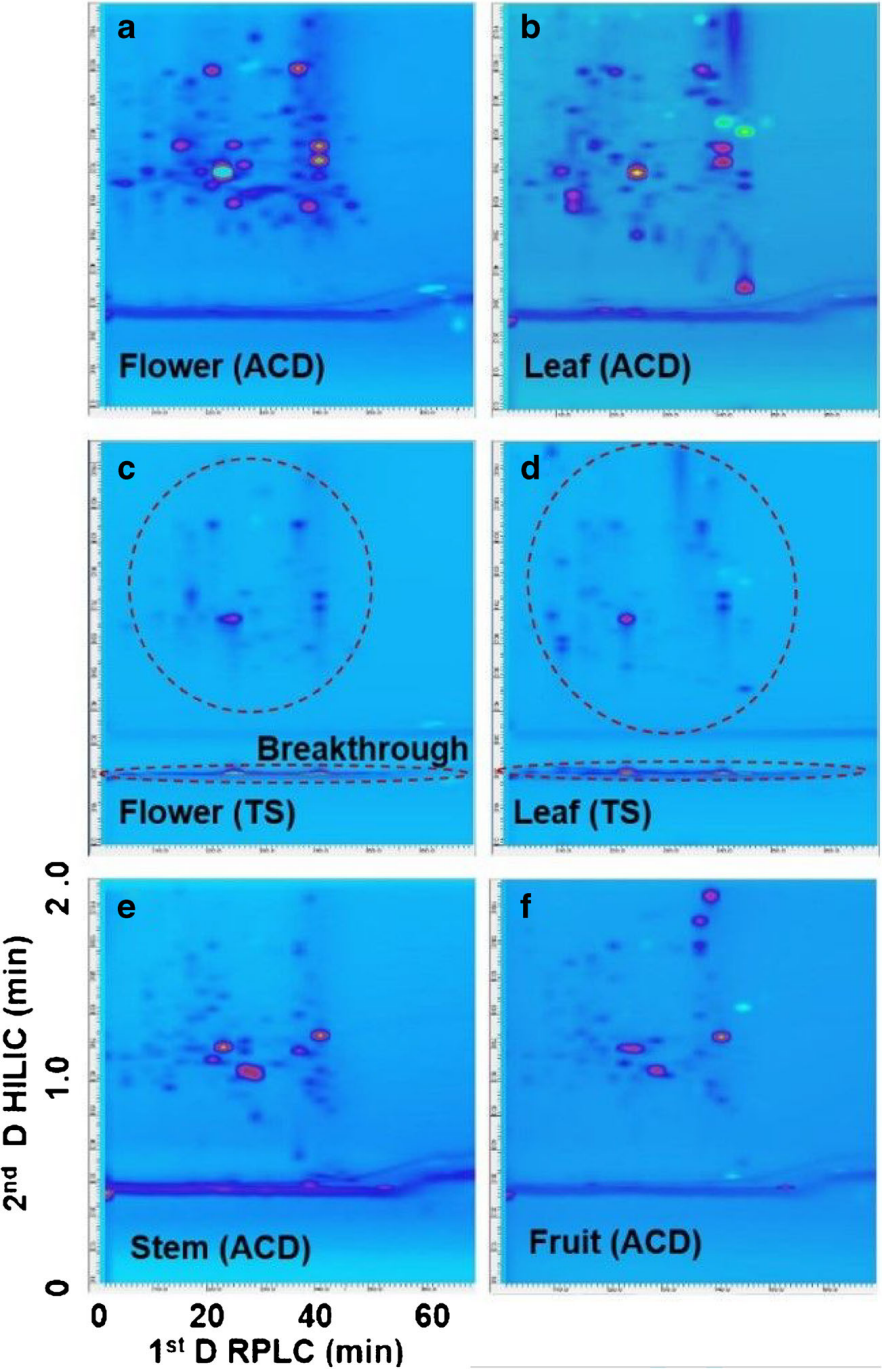
Contour plots of RPLCxHILIC with ACD modulation or with TS modulation for different parts of *Buddleja davidii.* Flower with ACD modulation (**a**), leaf with ACD modulation (**b**), leaf with TS modulation (**c**), flower with TS modulation (**d**), stem with ACD modulation (**e**), fruit with ACD modulation (**f**)

With the optimized ^1^D RP gradient and ^2^D HILIC shift gradient, the separation of the other four parts of *Buddleja davidii* including flower, leaf, stem, and fruit were performed on the RPLCxHILIC system with ACD modulation. The results are shown in Fig. 5a, b, e, and f. Due to the high-efficiency dilution of the transferred fraction, the constituents of the *Buddleja davidii* extracts were well separated into narrow and symmetric peaks over the ^2^D analysis. Compared to Fig. 5a, b, and f, a serious breakthrough could be observed in Fig. 5e. This result indicates that there are many homophilic compounds existing in the stem, which is similar to the result obtained in root separation. The results shown in Fig. 5a and b also indicate that more polar compounds with high concentration could be observed in the flowers and leaves than in other parts, which also implies that the metabolic profiles and, therefore, the metabolic activities of flowers and leaves are more vigorous than in other parts.

To demonstrate the advantage of the ACD modulation, the separation of the flower and leaf extract was also performed with TS modulation under the optimized gradient, the separation results are shown in Fig. 5c and d. Compared to the separation shown in Fig. 5a and b, without ACD modulation much less peaks could be separated and they presented lower peak intensities. This negative effect occurred because of the no-dilution of the fraction in the TS modulation which resulted in a considerable breakthrough in the dead volume. The solvent in the transferred fraction (high content of water) was a very strong elution solvent for the ^2^D HILIC separation, which prevents a focussing effect of the analytes on the head of the ^2^D HILIC column. In conclusion, the ACD modulation provides much better flexibility than the TS modulation for the dilution of the transferred fraction and the achievement of the re-focusing and ^2^D separation.

### Constituents analysis for the root of *Buddleja davidii* by RPLCxHILIC-Q-TOF MS

#### The advantage of RPLCxHILIC coupled to MS with ACD modulation

For further evaluation of the established RPLCxHILIC method, two 2D-LC configurations with and without ACD modulation were coupled to DAD and Q-TOF MS detectors to study the chemical constituents in the *Buddleja davidii* root. The high effective separation power provided by the ACD modulation facilitates the online identification of the constituents in root by coupling 2D-LC to high-resolution mass spectrometry. The results are shown in ESM Fig. S3. Figs. S3A and S3E show the UV contour plots for both measurements. As previously discussed, serious breakthrough could be observed at the dead volume without ACD modulation (ESM Fig. S3A), and much more improvement was achieved with ACD modulation (ESM Fig. S3E). The measurement without ACD modulation showed that only a few peaks could be well retained and observed in the 2D contour plot, which means that most constituents were eluted with the transferred fraction solvent in the ^2^D system without retention on the ^2^D HILIC stationary phase.

The same result could be obtained from the extracted ion chromatogram (EIC) contour plots in ESM Fig. S3. As observed from the EIC contour plots, with ACD modulation (ESM Figs. S3F, G and H), the spots show very good shapes and a clear background without any breakthrough, which can be attributed to the sufficient dilution of the transferred fraction. This leads to highly focus of amphiphilic analytes on the head of the ^2^D HILIC column. In contrast, with TS modulation, a significant peak splitting and fronting were found in the EIC contour plots (Figures S3B, C and D). It indicated that the amphiphilic compounds were divided in the ^2^D analysis into a non-retained portion and a retained portion. The compounds from the non-retained portion were quickly eluted together with the high water content solvent from the transferred fraction, forming a breakthrough peak in the dead volume due to a scarce dilution during the modulation. However, in the retained portion, part of the amphiphilic compounds presented relative separation in the ^2^D analysis probably due to an inter-diffusion effect in the loop between the transferred fraction and the ^2^D mobile phase, that allowed a partial focusing in the ^2^D column.

Nevertheless, the insufficient dilution produced by the inter-diffusion in the loop still resulted in serious peak fronting. As a serious consequence, the existence of the splitting peaks at dead volume might lead to a misunderstanding on the number of isomers, as shown in the EIC contour plot in ESM Figs. S3B, C and D, which could be considered as double when TS modulation is applied. Those results indicate that enough dilution of the transferred faction in high orthogonality 2D-LC is necessary to achieve proper separation and true MS chromatogram, avoiding misinformation.

The results of the EIC contour plots with ACD modulation demonstrate that the coupling of the proposed 2D-LC system with high-resolution mass spectrometry could provide high-quality MS information for the online identification of chemical components from complex samples.

#### Qualitative analysis of constituents in the roots of *Buddleja davidii*

The MS and MS/MS measurements of the root extract were performed with the optimized 2D-LC-UV-MS method. The general method for the identification of compounds in the sample mixture was carried out by comparing the detected molecular ion and MS/MS fragment ions with the reported information in the literature. Therefore, the reported researches about *Buddleja davidii* were carefully reviewed. Firstly, the comparison of the theoretically calculated molecular ion masses of the reported compounds with the detected molecular ion masses obtained by Q-TOF was done. Once the detected mass value was the same as the calculated ion mass of the reported compounds, it was postulated that the same compound was detected in the prepared sample. However, without MS/MS information and only with the sum formula is hardly possible to carry out an identification due to a multitude of possible isobaric compounds. Therefore, MS/MS analysis was performed. Even with MS/MS data, uncharacteristic fragmentation is often difficult to interpret and, besides, the differentiation between isomeric compounds is not possible. Here, the combination of ion mobility with MS could be helpful [22].

Due to the lack of previous information about the chemical composition of this plant, only a few compounds with defined structures were found in the reported work. To reduce this problem, another strategy was developed to characterize as much *Buddleja davidii* compounds separated by the 2D-LC-MS analysis as possible. According to the principles of biology, the plants with a genetic relationship usually have similar metabolic networks, resulting in similar chemical constituents. Based on this, in addition to the reported information of the compounds in *Buddleja davidii*, the components with defined structure in various species of the genus *Buddleja* were collected to extend the candidate compounds. As shown in ESM Fig. S1, in the literature several compounds of the genus *Buddleja* were described and these were considered possible candidates for *Buddleja davidii* [13–18, 23–29]. The accurate molecular ion masses [M-H]^−^ of each compound collected from the information about the *Buddleja* genus were calculated with Agilent MassHunter Qualitative Analysis Navigator software. Then the data were imported to the software LC Image. With this software, the contour plots can be easily generated, which allows the determination of a given molecular ion masses [M-H]^−^ existing in the huge total ion current (TIC) mass information. According to the calculated molecular ion masses [M-H]^−^ of the *Buddleja* described compounds, the EIC contour plots were extracted from the TIC contour plot.

After that, the compounds that were not found in the analysis were excluded. For example, two typical compounds present in the genus *Buddleja* have an *m/z* 283.0612 and 423.3632, which correspond to acacetin and δ-amyrone, respectively. The EIC of both compounds in the RPxHILIC analysis of the root is shown in ESM Fig. S4. In the EIC contour plot of *m/z* 283.0612, a clear spot was observed, which means that this compound is presented with a relative high concentration. In contrast, in the contour plot of the *m/z* 423.3632, there is no clear spot, but a complex background was observed, which means that the ion with *m/z* 423.3632 was not present in *Buddleja davidii* root or its intensity was too low to be detected. Thus, this ion would be ignored in this study.

Obviously, in the TIC, many more ions existed than those we screened with the above method. In order to find out more ions with relatively high concentrations, further effort was done with the LC image software by comparing the UV and TIC contour plots. On one hand, the positions of the signals in the TIC contour plot were compared with the spots in the UV contour plot, and carefully examined to find out new ions, and then the EIC contour plot was generated and saved in LC image software. On the other hand, the spots observed in the TIC contour plot, but not in the UV contour plot, were also checked. After that, the ions in the EIC contour plot were further selected and excluded according to the method shown in ESM Fig. S4. Considering the molecular mass and the MS/MS fragments, these compounds were also tentatively identified.

Finally, EIC contour plots, which were screened by the previous three methods, were merged. As shown in ESM Fig. S5, some EIC contour plots contained one spot, for example in the *m/z* 905.2727 (ESM Fig. S5 left panel). Other EIC contour plots might contain several spots for the same mass, which indicated the existence of multiple isomers such as *m/z* 637.2148 (ESM Fig. S5 right panel). For both situations, the molecular ions and corresponding MS/MS fragments, once detected, were collected and then listed in Table 1. In total, 45 compounds including isomers have been found in the *Buddleja davidii* root sample. However, due to scarce available information about the chemical composition of this plant, some ions could not be identified accurately by comparing to the detected ions with the reported works.

**Table 1:** MS identification of the constituents found in the RPxHILIC-DAD-QTOF MS analysis of the root sample of *Buddleja davidii*

Nr.	Retention time (min)	[M-H]^−^	Experimental mass	Theoretical mass	Error (ppm)	Chemical formula	Fragments MS/MS	Identification	Ref.
1	41.26	283.0632	284.0710	284.0685	8.9767	C_16_H_12_O_5_	107, 151, 211, 239, 268	Acacetin	[27]
2	12.74	329.0883	330.0961	330.0951	3.1506	C_14_H_17_O_9_	108, 152, 197	Vanillic acid glucoside	[28]
3	46.59	329.2355	330.2433				139, 171, 211, 226, 292, 319		
4	7.9	341.1107	342.1185	342.1162	6.7521	C_12_H_22_O_11_	101, 113, 119	Sucrose	[15]
5	60.6	375.0885	376.0974				278, 331, 345, 360		
6	57.7	459.2535	460.2613			C_30_H_35_O_4_	125, 152, 181, 199, 277	Dihydrobuddledin A derivative	
7	9.35	461.1688	462.1766			C_23_H_26_O_10_	101, 113, 135, 153, 297, 315	Verbacoside derivate	
8	15.29	476.1838	477.1916				101, 113, 134,160, 175, 193		
9	13.55	487.1476	488.1554	488.153	4.9779	C_21_H_28_O_13_	135, 161, 179	Cistanoside F	[27]
10	27.09	497.1736	498.1814				108, 123, 152, 167	Vanillic acid derivate	
11	11.63	503.1674	504.1752				101, 113, 131, 161, 221		
12	29.07	521.1719	522.1797				121, 148, 160, 175,193, 341		
13	24.84	523.1848	524.1926				134, 160, 175, 193		
14	36.77	523.1861	524.1939				134, 160, 175, 193		
15	37.07	523.1861	524.1939				134, 160, 175, 193		
16	13.54	529.1586	530.1664				113, 133, 161		
17	15.21	529.1589	530.1667				113, 133, 161		
18	19.17	551.2152	552.2230				101, 167, 220, 235, 311, 326, 356, 371		
19	25.04	551.2159	552.2237				101, 167, 220, 235, 311, 326, 356, 371		
20	41.26	591.1732	592.1810	592.1792	3.065	C_28_H_32_O_14_	284, 268	Linarin	[17]
21	27.24	623.2008	624.2086	624.2054	5.1265	C_29_H_36_O_15_	113, 135, 161, 461	Verbascoside/Forsythoside A/Acteoside/Cis-acteoside/Isoacteoside	[29]
22	23.21	623.201	624.2088	624.2054	5.4469	C_29_H_36_O_15_	113, 135, 161, 461	Verbascoside/Forsythoside A/Acteoside/Cis-acteoside/Isoacteoside	[29]
23	41.26	637.1818	638.1896				113, 135, 160, 17, 193, 461, 161		
24	33.11	637.2170	638.2248	638.2211	5.8757	C_30_H_38_O_15_	113, 135, 160, 175, 193, 461, 161	Jionoside D/Plantainoside C	[14]
25	35.16	637.2178	638.2256	638.2211	7.1292	C_30_H_38_O_15_	113, 135, 160, 175, 193, 461, 161	Jionoside D/Plantainoside C	[14]
26	17.35	639.1970	640.2048	640.2003	7.0056	C_29_H_36_O_16_	113, 135, 151, 161, 179	Campneoside II/Isocampneoside II	[15, 26]
27	39.05	651.2340	652.2418				113, 134, 160, 175	Isomartynoside/Martynoside)/crocetin mono- gentibiosyl ester	[28]
28	13.84	665.2185	666.2263				101, 113, 143,179, 221, 383		
29	17.95	665.2223	666.2301				101, 113, 143,179, 221, 383		
30	19.78	665.2262	666.234				101, 113, 143,179, 221, 383		
31	9.2	671.2388	672.2466				101, 167, 297, 549, 630, 653		
32	51.54	675.3616	676.3694			C_33_H_55_O_14_	119, 179, 235, 277, 397, 415	Dihydrobuddledin A derivate	[15]
33	41.1	701.2499	702.2577				193, 279, 294, 323, 341, 353,671		
34	37.22	719.261	720.2688				134, 150, 165, 193, 341, 523		
35	46.586	735.2538	736.2616	736.2579	5.1205	C_35_H_44_O_17_		Acetylmartynoside A	[14]
36	19.477	755.243	756.2508	756.2477	4.1521	C_34_H_44_O_19_	135,161,179, 429, 447, 593	Forsythoside B/angoroside A/Hebeoside	[15, 24, 25]
37	27.397	769.2594	770.2672	770.2633	5.0502	C_35_H_46_O_19_	135, 161, 175, 193, 447, 575, 593, 607	Poliumoside	[15]
38	25.417	769.2607	770.2685	770.2633	6.7380	C_35_H_46_O_19_	135, 161, 175, 193, 447, 575, 593, 607	Poliumoside	[15]
39	23.437	769.2616	770.2694	770.2633	7.9064	C_35_H_46_O_19_	135, 161, 175, 193, 447, 575, 593, 607	Poliumoside	[15]
40	29.377	783.2739	784.2817	784.279	3.4937	C_36_H_48_O_19_	125, 160, 175, 193, 607	Angoroside C	[25]
41	34.97	783.2747	784.2825	784.279	4.5137	C_36_H_48_O_19_	125, 160, 175, 193, 607	Angoroside C	[25]
42	35.24	783.2755	784.2833	784.279	5.5337	C_36_H_48_O_19_	125, 160, 175, 193, 607	Angoroside C	[25]
43	17.574	785.2532	786.2610	786.2583	3.5294	C_35_H_46_O_20_	161, 179, 383, 461, 623	Echinacoside	[26]
44	37.296	797.2887	798.2965				101, 175, 193, 457, 475, 621		
45	33.336	905.2746	906.2824				161, 575, 593, 743		

## Conclusion

In this study, a recent developed RPLCxHILIC system with at-column dilution (ACD) modulation was applied for a comprehensive analysis of constituents in *Buddleja davidii*. With ACD modulation, the incompatibility of high orthogonal 2D-LC systems could be successfully overcome. Meanwhile, the flexible adjustment of the dilution of the transferred fraction supported the advantages of the RPLCxHILIC coupling for an excellent separation of amphiphilic compounds with a wide range of polarity. The results demonstrated the potential of a 2D-LC system with ACD modulation for wide applications in the herbal medicine analysis. With this method, a broad separation of the metabolites present in *Buddleja davidii* was achieved. However, more information about the chemical composition of the separated compounds should be done to obtain comprehensive information about the metabolic profile of this herbal medicinal. Nevertheless, the detailed MS and MS/MS data of some detected ions with relative high intensities can be considered as a good reference for further studies.

## Electronic supplementary material

ESM 1 (PDF 663 kb)
