# Supplementary material for: Application of the new at-column dilution (ACD) modulator for the two-dimensional RP×HILIC analysis of Buddleja davidii
Source: Anal Bioanal Chem. 2020 Jan 21;412(7):1483–95. doi: 10.1007/s00216-020-02392-3 (PMC7026260; doi:10.1007/s00216-020-02392-3)
Supplement: Supplementary file 1 — (PDF 663 kb) [file 216_2020_2392_MOESM1_ESM.pdf]

Analytical and Bioanalytical Chemistry

Electronic Supplementary Material

Application of the new at-column dilution (ACD) modulator for the two-dimensional RP×HILIC analysis of *Buddleja davidii*

Yingzhuang Chen, Lidia Montero, Jiang Luo, Junjie Li, Oliver J. Schmitz

Content

Page 2	Figure S1. Scheme of the LC×LC system with trap column-based fixed solvent modulation (FSM).
Page 3	Figure S2. Constituents in genus *Buddleja*
Page 7	Figure S3. Contour plots for 2D-LC analysis of *Buddleja davidii* root.
Page 7	Figure S4. EIC contour plot of acacetin and δ-amyrone
Page 7	Figure S5. EIC contour plots with m/z 637.2148 and 905.2727
Page 8	Table S1. Optimization of the 1D gradient

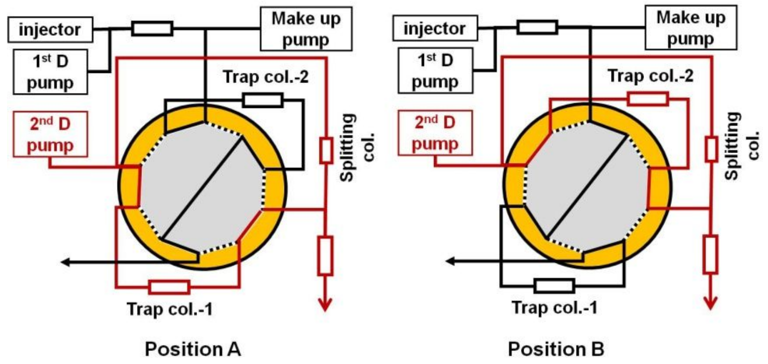

Fig. S1 Scheme of the LC×LC system with trap column-based fixed solvent modulation (FSM)

Fig. S2

a: terpenes

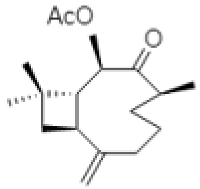

dihydrobuddledin A

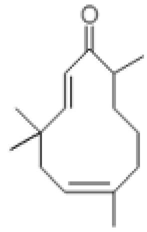

buddledones A

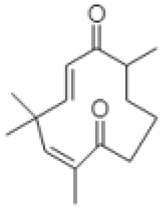

buddledones B

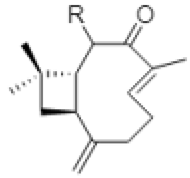

buddledins A(1),B(2),C(3)

	R
1	OAc
2	OH
3	H

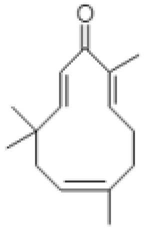

Zerumbone

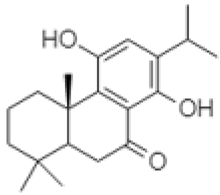

11,14-dihydroxy-8,11,13-abietatrien-7-one

b: phenylethanoids

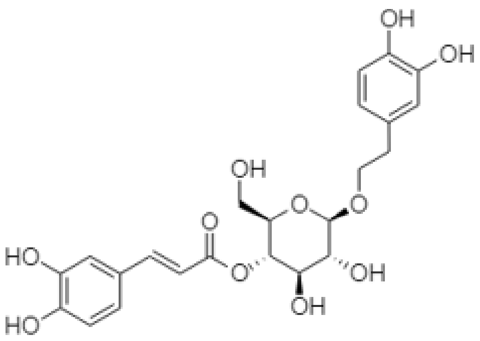

Calceolarioside A

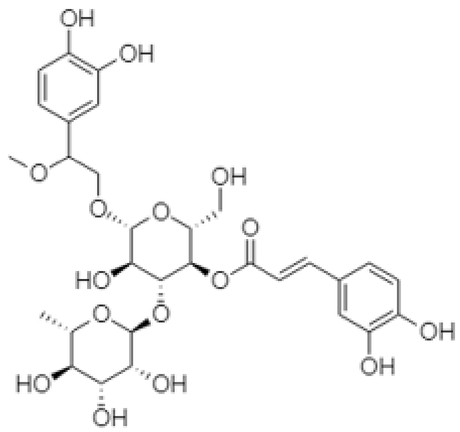

Campneoside

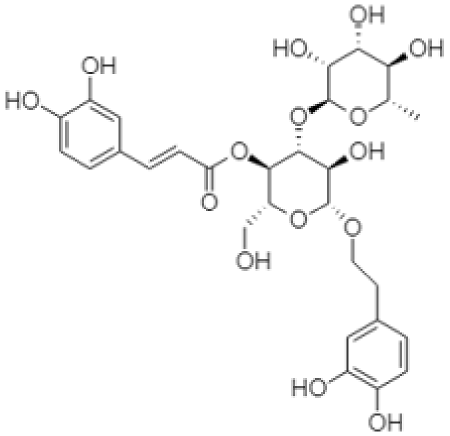

Verbascoside

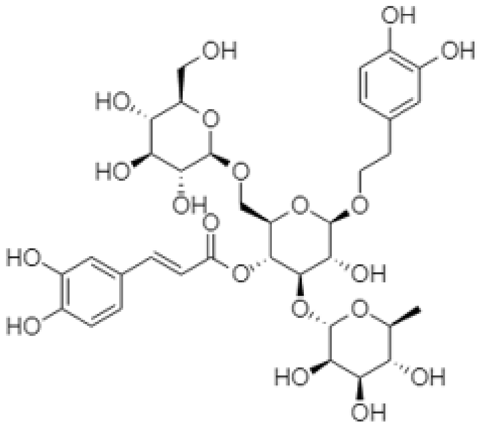

Echinacoside

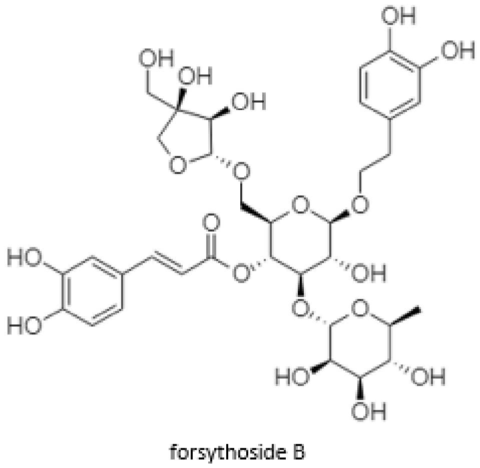

forsythoside B

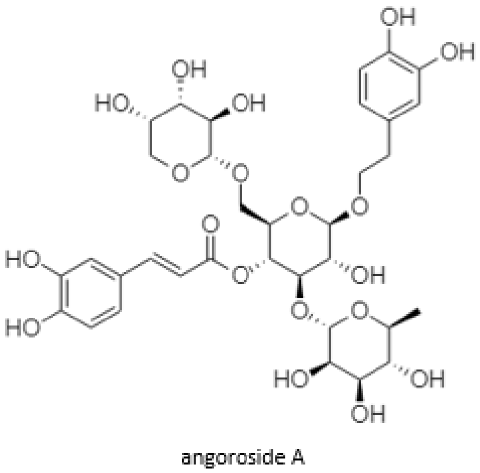

angoroside A

c: phenylpropanoid glycoside

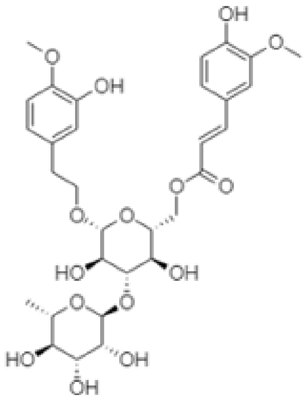

Isomartynoside

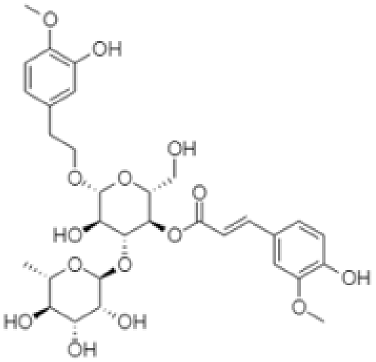

Martynoside

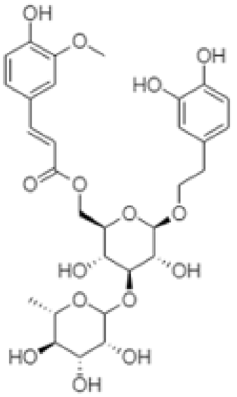

Plantainoside C

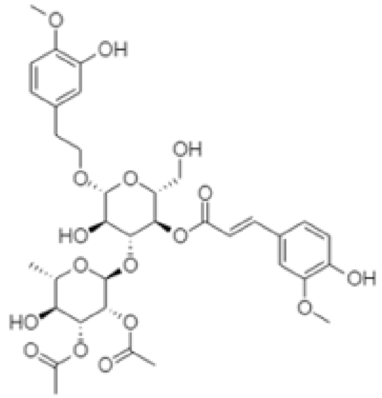

Acetylmartynoside A

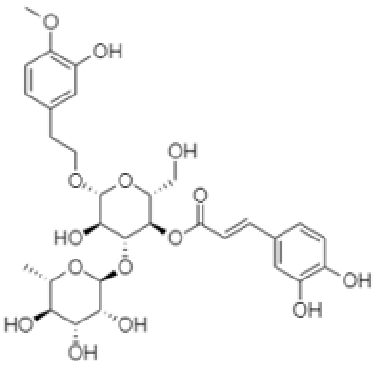

Jionoside D

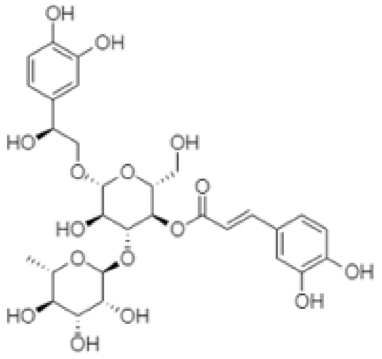

Campneoside II

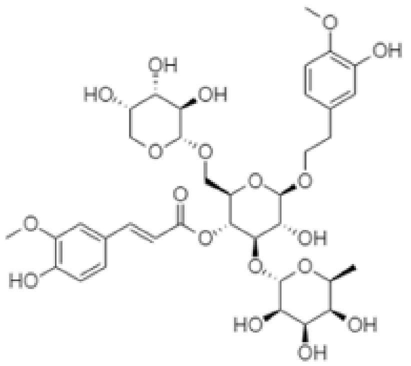

Angoroside C

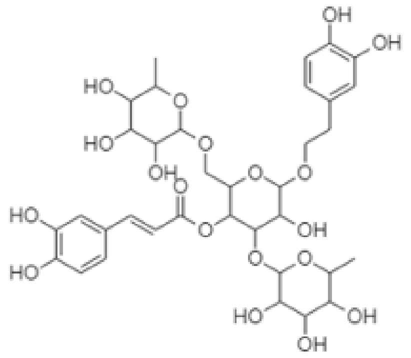

Poliumoside

d: miscellaneous

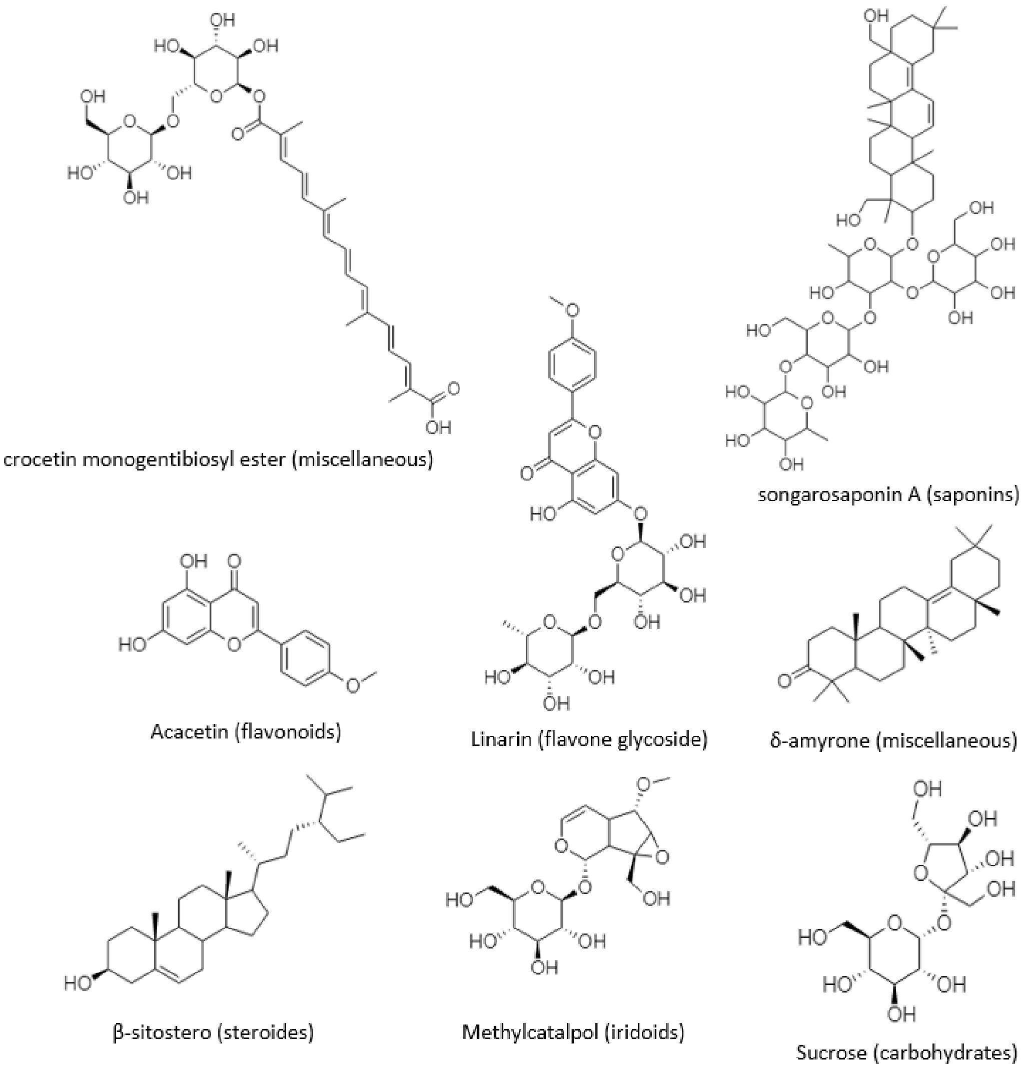

Fig. S2 Compounds in groups of terpenes (a), phenylethanoids (b), phenylpropanoid glycosides (c) and miscellaneous, flavonoids, saponins, steroids, iridoids and carbohydrates (d). [1-13]

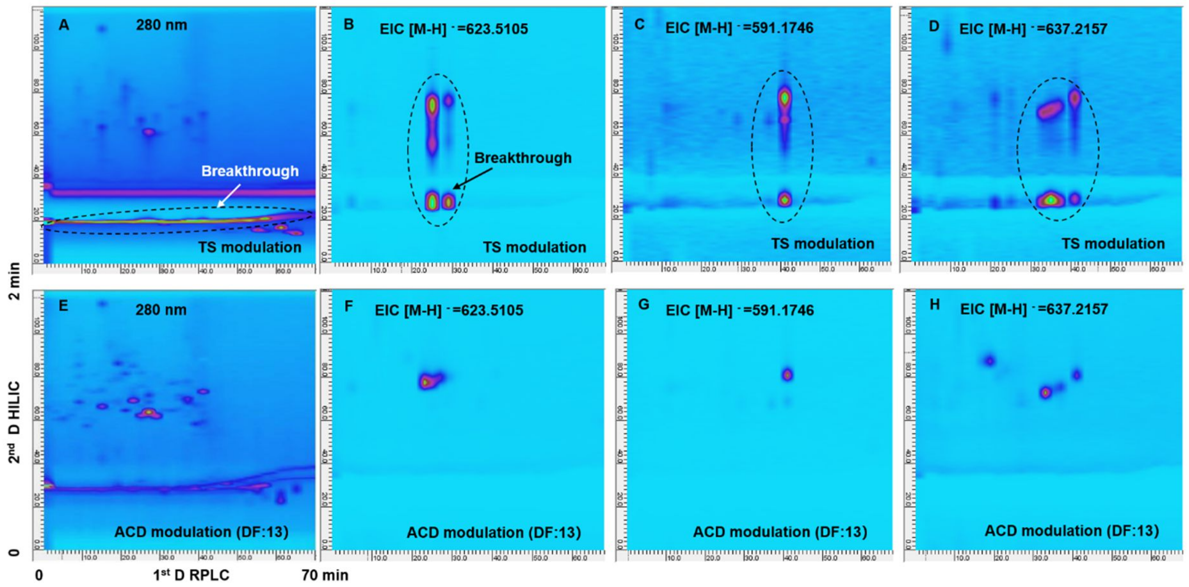

Fig. S3 Counter plots for 2D-LC analysis of *Buddleja davidii* root. UV contour plot with TS modulation (A), UV contour plot with ACD modulation (E), and EIC contour plot for selected ions with TS and ACD modulation, $[M-H]^-$ =623.5015 (B and F), $[M-H]^-$=591.1746 (D and G), $[M-H]^-$=637.2157 (C and H), respectively.

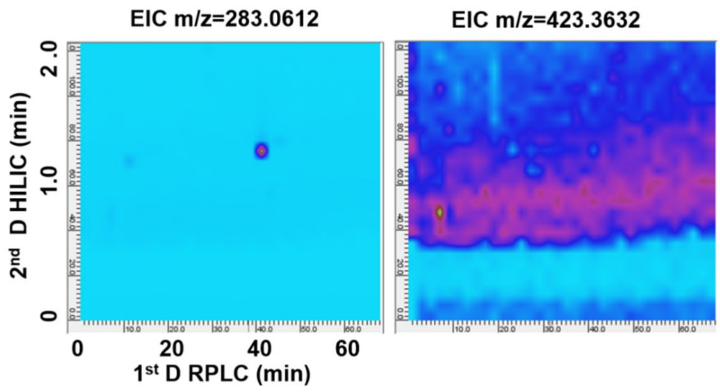

Fig. S4 EIC contour plot for theoretical molecular ions of acacetin (*m/z* 283.0612) and δ-amyrone (*m/z* 423.3632)

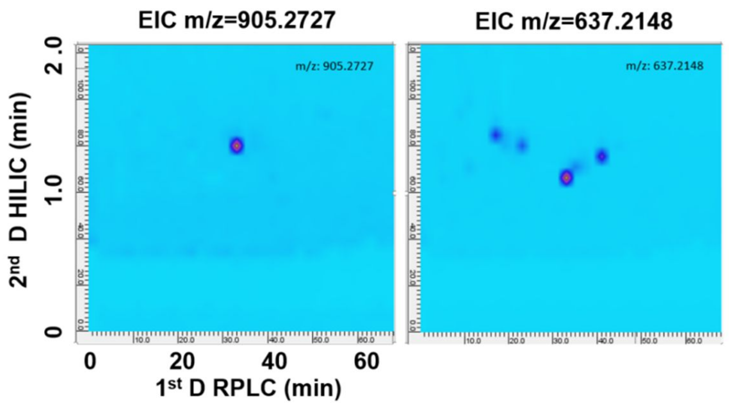

Fig. S5 EIC contour plots of *m/z* 905.2727 and 637.2148

Table S1 Optimization of the ^{1}D gradient with acetonitrile content (B)

Gradient									
**a**	Time[min]	0	5	56					
	B[%]	10	10	92					
**b**	Time[min]	0	3	30	35	50	52	56	
	B[%]	10	20	25	100	100	10	10	
**c**	Time[min]	0	3	25	30	40	50	52	56
	B[%]	10	20	20	70	100	100	10	10
**d**	Time[min]	0	3	25	45	50	52	56	
	B[%]	10	20	20	100	100	10	10	
**e**	Time[min]	0	3	20	36	50	63	65	70
	B[%]	10	20	20	45	100	100	10	10

References

1. Ahmad M, Sticher O. Isolation of acacetin-7-O-rutinoside and martynoside from *Buddleja davidii*. J Chem Soc Pakistan 1988;10:117-23.
2. Duff RB, Bacon JS, Mundie CM, Farmer VC, Russell JD, Forrester AR. Catalpol and methylcatalpol: naturally occurring glycosides in Plantago and Buddleia species. Biochem J 1965;96:1-5.
3. Endo K, Takahashi K, Abe T, Hikino H. Structure of Forsythoside-B, an antibacterial principle of Forsythia-Koreana Stems. J Heterocyclic Chem. 1982;19:261-4.
4. Feng X, Wang X, Liu Y, Di X. Linarin inhibits the acetylcholinesterase activity in-vitro and ex-vivo. Iran J Pharm Res. 2015;14:949-54.
5. Gross G-A, Sticher O, Calis I. Phenylpropanoid glycosides isolated from *Scrophularia scopolii*. Phytochemistry. 1987;26:2057-61.
6. Hoult JRS, Moroney MA, Payá M Actions of flavonoids and coumarins on lipoxygenase and cyclooxygenase. Method. Enzymol., Academic Press,1994; 234: 443-54.
7. Kitagawa S, Tsukamoto H, Hisada S, Nishibe S. Studies on the Chinese crude drug "*Forsythiae fructus*."vii. A new caffeoyl glycoside from *Forsythia viridissima*. Chem Pharm Bull. 1984;32:1209-13.
8. Liao Y, Houghton PJ, Hoult JRS. Novel and known constituents from buddleja species and their activity against leukocyte eicosanoid generation. J Nat Prod 1999;62:1241-5.
9. Matsuda H, Cai H, Kubo M, Tosa H, Iinuma M. Study on anti-cataract drugs from natural sources. II. Effects of buddlejae flos on in vitro aldose reductase activity. Biol Pharm Bull. 1995;18:463-6.
10. Pfander H, Wittwer F. Carotinoid-Glycoside. 3. Mitteilung. Untersuchungen zur carotinoidzusammensetzung im safran. Helv Chim Acta. 1975;58:2233-6.
11. Vertuani S, Beghelli E, Scalambra E, Malisardi G, Copetti S, Toso RD, Baldisserotto A, Manfredini S. Activity and stability studies of verbascoside, a novel antioxidant, in dermo-cosmetic and pharmaceutical topical formulations. Molecules. 2011;16.
12. Yamamoto A, Nitta S, Miyase T, Ueno A, Li-Jun W. Phenylethanoid and lignan-iridoid complex glycosides from roots of *Buddleja davidii*. Phytochemistry. 1993;32:421-5.
13. Zimin L, Zhongjian J. Phenylpropanoid and iridoid glycosides from *Pedicularis striata*. Phytochemistry. 1991;30:1341-4.
